# Diabetes Is the Main Factor Accounting for Hypomagnesemia in Obese Subjects

**DOI:** 10.1371/journal.pone.0030599

**Published:** 2012-01-24

**Authors:** Albert Lecube, Juan Antonio Baena-Fustegueras, José Manuel Fort, Dolors Pelegrí, Cristina Hernández, Rafael Simó

**Affiliations:** 1 CIBER de Diabetes y Enfermedades Metabólicas Asociadas (CIBERDEM), Instituto de Salud Carlos III (ISCIII), Diabetes and Metabolism Research Unit, Institut de Recerca i Hospital Universitari Vall d'Hebron (VHIR), Endocrinology Department, Universitat Autònoma de Barcelona, Barcelona, Spain; 2 Endocrinology Surgery Unit, General Surgery Department, Hospital Universitari Vall d'Hebron, Universitat Autònoma de Barcelona, Barcelona, Spain; 3 Biochemistry Department, Hospital Universitari Vall d'Hebron, Universitat Autònoma de Barcelona, Barcelona, Spain; University of Tor Vergata, Italy

## Abstract

**Objective:**

Type 2 diabetes (T2DM) and obesity are associated with magnesium deficiency. We aimed to determine whether the presence of type 2 diabetes and the degree of metabolic control are related to low serum magnesium levels in obese individuals.

**Methods:**

A) Case-control study: 200 obese subjects [50 with T2DM (cases) and 150 without diabetes (controls)] prospectively recruited. B) Interventional study: the effect of bariatric surgery on serum magnesium levels was examined in a subset of 120 obese subjects (40 with type 2 diabetes and 80 without diabetes).

**Results:**

Type 2 diabetic patients showed lower serum magnesium levels [0.75±0.07 *vs.* 0.81±0.06 mmol/L; mean difference −0.06 (95% CI −0.09 to −0.04); p<0.001] than non-diabetic patients. Forty-eight percent of diabetic subjects, but only 15% of non-diabetic subjects showed a serum magnesium concentration lower than 0.75 mmol/L. Significant negative correlations between magnesium and fasting plasma glucose, HbA1c, HOMA-IR, and BMI were detected. Multiple linear regression analysis showed that fasting plasma glucose and HbA1c independently predicted serum magnesium. After bariatric surgery serum magnesium increased only in those patients in whom diabetes was resolved, but remain unchanged in those who not, without difference in loss weight between groups. Changes in serum magnesium negatively correlated with changes in fasting plasma glucose and HbA1c. Absolute changes in HbA1c independently predicted magnesium changes in the multiple linear regression analysis.

**Conclusions:**

Our results provide evidence that the presence of diabetes and the degree of metabolic control are essential in accounting for the lower levels of magnesium that exist in obese subjects.

## Introduction

Magnesium is the fourth most abundant cation in the human body and the second most profuse intracellular cation. It is an important cofactor in a number of key enzymatic reactions and appears to play an important role in glucose metabolism and insulin homeostasis. In recent years, increasing evidence has appeared suggesting an association between magnesium deficiency and type 2 diabetes mellitus (T2DM) [1]–[5]. In the *Atherosclerosis Risk in Communities Study*, Caucasian men with serum magnesium <0.58 mmol/L had a two-fold increase in incidence of T2DM compared with those with a magnesium concentration >0.78 mmol/L [1]. Furthermore, the available data also indicates that low dietary magnesium intake may be an independent risk factor for the development of T2DM [1], [3]. In this regard, the *Health Professionals Follow-Up Study* and the *Nurse's Health Study* showed that subjects in the highest quintile of magnesium intake had a 33% lower risk of developing T2DM than those in the lowest quintile of magnesium intake [3]. Finally, magnesium supplementation in subjects with T2DM resulted in an improvement of insulin sensitivity and metabolic control [2]. However such findings have not been reported consistently in other trials [6].

The mechanisms whereby hypomagnesemia may induce or worsen existing diabetes are not well understood. Nonetheless, it seems that both insulin secretion and insulin action can be affected [7]. Alternatively, some studies performed in non-morbidly obese subjects showed that insulin resistance and chronic hyperglycemia might contribute to the development of hypomagnesemia [8], [9]. Because insulin has been implicated in enhancing renal magnesium reabsortion, insulin deficiency or resistance could promote urinary magnesium excretion [7], [10]. Therefore, hypomagnesemia seems to be a contributing factor for T2DM development but type 2 diabetes could also be involved in low magnesium levels found in the diabetic population.

Some cross-sectional studies have found that obese individuals have lower circulating magnesium concentrations than healthy subjects [11]. However, it is unknown whether the lower levels of magnesium found in obese subjects are related to the presence of associated diabetes rather than to obesity itself. Since bariatric surgery by the Roux-en-Y gastric bypass (RYGBP) is associated with high percentages of T2DM resolution [12], it represents a good model for testing the hypothesis that T2DM is the main factor accounting for the low magnesium levels that exist in obese patients.

On this basis, the overall aim of this study was to determine whether the presence of T2DM and the degree of metabolic control are related to low serum magnesium levels in obese individuals. To shed light to this issue we performed two types of studies: 1) A case-control study in order to compare serum magnesium levels between T2DM and non-diabetic obese subjects closely matched by BMI, age and gender; 2) An interventional study in order to elucidate whether those patients in whom the surgical procedure normalized blood glucose levels also showed a significant increase in serum magnesium levels.

## Materials and Methods

### Ethics statement

Informed written consent was obtained from all participants and the study was approved by the hospital's human ethics committee (Hospital Universitari Vall d'Hebron).

### Design of the study and description of study population

#### Cross sectional case-control study

In this study we have investigated the association between serum magnesium levels and T2DM in morbidly obese subjects, following the “Strengthening the Reporting of Observational Studies in Epidemiology” (STROBE) guidelines for reporting case-control studies [13]. We used the following formula for the sample size calculation:
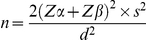
where the alpha level was set at p<0.05 (Zα) and the minimum acceptable power level was considered to be 0.80 (Zβ). *s*: is the population standard deviation of serum magnesium in obese subjects detected in a previous study [14]. *d:* the postulated effect size (we considered clinically significant a difference in serum magnesium between the two groups of 0.04 mmol/L). *n:* is the sample size.
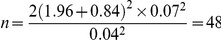



On this basis, a total of fifty consecutive morbidly obese type 2 diabetic subjects without associated complications attending the outpatient Obesity Unit of a university hospital (Hospital Universitari Vall d'Hebrón, Barcelona, Spain) were recruited for the study over a 12-month period (cases). We aimed to select 3 controls for every case and, consequently, one-hundred and fifty non-diabetic subjects attending the same Obesity Unit served as a control group. Controls were individually matched to cases by BMI, age and gender.

None of the participants had a positive clinical history of malignancy, chronic diarrhea, chronic or acute renal failure, alcohol intake (≥30 g per day), nor abuse of any kind of drug. Subjects receiving magnesium supplementation or treated with drugs known to modify magnesium metabolism (such as diuretics, thyroxine, lithium and/or calcium antagonist) were excluded from the study. None of the women were pregnant and no patient with type 1 diabetes was included. According to previous studies, hypomagnesemia was defined as a serum magnesium concentration ≤0.75 mmol/L [5], [15], [16]. T2DM was defined according to the criteria recommended by the Expert Committee on the Diagnosis and Classification of Diabetes.

#### Interventional study: Surgical induced weight loss study

The effect of bariatric surgery on serum magnesium levels was examined in 40 subjects from the type 2 diabetic group who met the eligibility criteria for gastrointestinal surgery established by the guidelines of the National Institutes of Health Consensus Conference. In these patients a RYGBP surgery was performed over a 18-month period. Briefly, the laparoscopically RYGBP was performed using 5 trocars placed in a standard fashion for laparoscopic upper gastrointestinal surgery. Carbon dioxide pneumoperitoneum (15 mmHg) was created using the Veress needle technique. A 30-mL gastric pouch was then tailored around a regular Fouchet tube. To create the jejunojejunostomy, both the greater omentum and transverse colon were passed to the upper abdomen, and the jejunum was transected at 60 cm from the ligament of Treitz. The Roux limb was then measured 150 cm distally, and a side-to-side anastomosis was created with the proximal jejunal limb. Finally, the end-to-side gastrojejunostomy was created. Eighty non-diabetic obese subjects who also underwent RYGBP surgery during the same period served as a control group. Controls were individually matched to cases by BMI, age and gender.

After surgery all patients were given the following diet: during the first ten days, a fluid diet of 810 kcal/day (45% carbohydrates, 33% protein, and 22% fat); between days 11 and 20, a triturated diet of 839 kcal/day (44% carbohydrates, 38% protein, and 18% fat); during the last ten days of the first month, a solid diet of 844 kcal/day (41% carbohydrates, 31% protein, and 28% fat); from the second months to the end of the study, all patients were placed on a maintenance diet of 825 kcal (44% carbohydrates, 34% protein, and 22% fat). No patient received magnesium supplementation.

The exclusion criteria were the same as that above mentioned for the case-control study. Patients were followed during a 6-month period and diabetes resolution was defined as the ability to discontinue all diabetes-related medications and maintain blood glucose and HbA1c levels within the normal range (≤5.5 mmol/L and ≤6.0%, respectively) [12]. Blood samples for general analyses and magnesium measurements were drawn at baseline 3 and 6 months after surgery.

### Laboratory assessment

All laboratory measurements were performed on fasting blood samples. Serum magnesium was measured by spectrophotometrical xylidyl blue method on Olympus AU2700 analyser using its own commercial kit (Olympus Diagnostics GmBH, Hamburg, Germany). The principle of the method depends on the formation of colored complex between magnesium ions with xylidyl blue in an alkaline medium. The laboratory coefficient of variation for magnesium, based on split samples, was 1.9%. Other biochemical parameters analyzed included measurement of plasma glucose, insulin, and HbA1c. These parameters were measured by standard laboratory techniques used in clinical chemistry laboratories. Insulin resistance was determined by the *homeostasis model assessment* [HOMA-IR = insulin (mU/L)×glucose (mmol/L)/22.5)]. HOMA-IR was restricted to individuals no currently taking antidiabetic drugs. Finally, serum 25-hydroxy vitamin D [25(OH)D] and intact parathyroid hormone (iPTH) were measured by a direct competitive chemiluminescense immunoassay (CLIA) (DiaSorin Inc., Stillwater, MN, USA) using the LIAISON Analyzer family. The means intra and interassay coefficient of variation for 25(OH)D were 4.5% and 7.6% respectively. The means intra and interassay coefficient of variation for iPTH were 3.8% and 7.9% respectively.

### Statistical analysis

Normal distribution of the variables was evaluated using the Kolmogorov-Smirnov test. Data were expressed either as the mean ± SD, median (range), or percentage. Given their skewed distribution, HOMA-IR data was logarithmically transformed to achieve a normal distribution. Comparisons between groups were performed using the unpaired Student *t* test for continuous variables, and the *χ* ^2^ test for categorical variables. The change of the variables compared with the baseline values were used for the analysis of treatment (bariatric surgery) effect for each group using Student-t test for paired data. The relationship between continuous variables was examined by the Pearson linear correlation test.

In the cross sectional case-control study a stepwise multiple linear regression analysis was performed in order to explore the variables independently related to serum magnesium. The variables included were age, BMI, fasting plasma glucose, HbA1c, and HOMA-IR. In the interventional study, a new stepwise multiple linear regression analysis was performed in order to explore the variables independently related to the absolute change of serum magnesium at 6 months. The variables included were BMI, age, and absolute changes in fasting plasma glucose and HbA1c.

All *p* values were based on a two-sided test of statistical significance. Significance was accepted at the level of *p*<0.05. Statistical analyses were performed with the SSPS statistical package (SPSS Inc, Chicago, Illinois).

## Results

### Cross sectional case-control study

The main clinical features and laboratory data of the study population according to the presence of T2DM are presented in Table 1. Diabetic patients showed lower serum magnesium concentrations in comparison with non-diabetic patients [0.75±0.07 vs. 0.81±0.06 mmol/L; mean difference −0.06 (95% CI −0.09 to −0.04); p<0.001]. Forty-eight percent of diabetic subjects showed a serum magnesium concentration lower than 0.75 mmol/L, but only fifteen percent of non-diabetic subjects (p<0.001) did so.

**Table 1:** The main clinical features and laboratory data of the study population from the cross sectional case-control study according to the presence of type 2 diabetes.

	Type 2 Diabetes	Non-diabetic	Mean difference (95%CI)	p
**n**	50	150	-	-
**Women, n (%)**	40 (80.0)	114 (76.0)	-	0.355
**Age (years)**	45.06±8.59	44.71±8.25	0.35 (−2.33 to 3.04)	0.796
**BMI (Kg/m^2^)**	46.45±5.87	46.30±5.46	0.15 (−1.60 to 1.94)	0.869
**Mg (mmol/L)**	0.75±0.07	0.81±0.06	−0.06 (−0.09 to −0.04)	<0.001
**Mg≤0.75 mmol/L, n (%)**	24 (48.00)	23 (15.33)	-	<0.001
**Fasting plasma glucose (mmol/L)**	8.73±3.47	5.60±1.16	3.12 (2.12 to 4.13)	<0.001
**HbA1c (%)**	7.60±1.87	5.76±0.56	1.84 (1.25 to 2.43)	<0.001
**Fasting insulin (mU/L)**	23.40±12.65	19.76±11.43	3.64 (−0.44 to 7.72)	0.080
**HOMA-IR** *	8.04 (0.84–38.88)	4.26 (0.93–23.46)	-	<0.001
**25(OH)D (nmol/L)**	46.05±23.92	47.85±25.05	−1.80 (−10.62 to 7.02)	0.688
**iPTH (ng/mL)**	67.06±28.29	69.45±22.30	−2.39 (−12.50 to 7.72)	0.640

Data are mean ± SD, median (range), and percentage. Mg: serum magnesium; BMI: body mass index; HOMA-IR: insulin resistance measured by the homeostasis model assessment; 25(OH)D: 25-hydroxy vitamin D; iPTH: intact parathyroid hormone.

*: Restricted to individuals without current hypoglycaemic medication.

**Figure 1:**
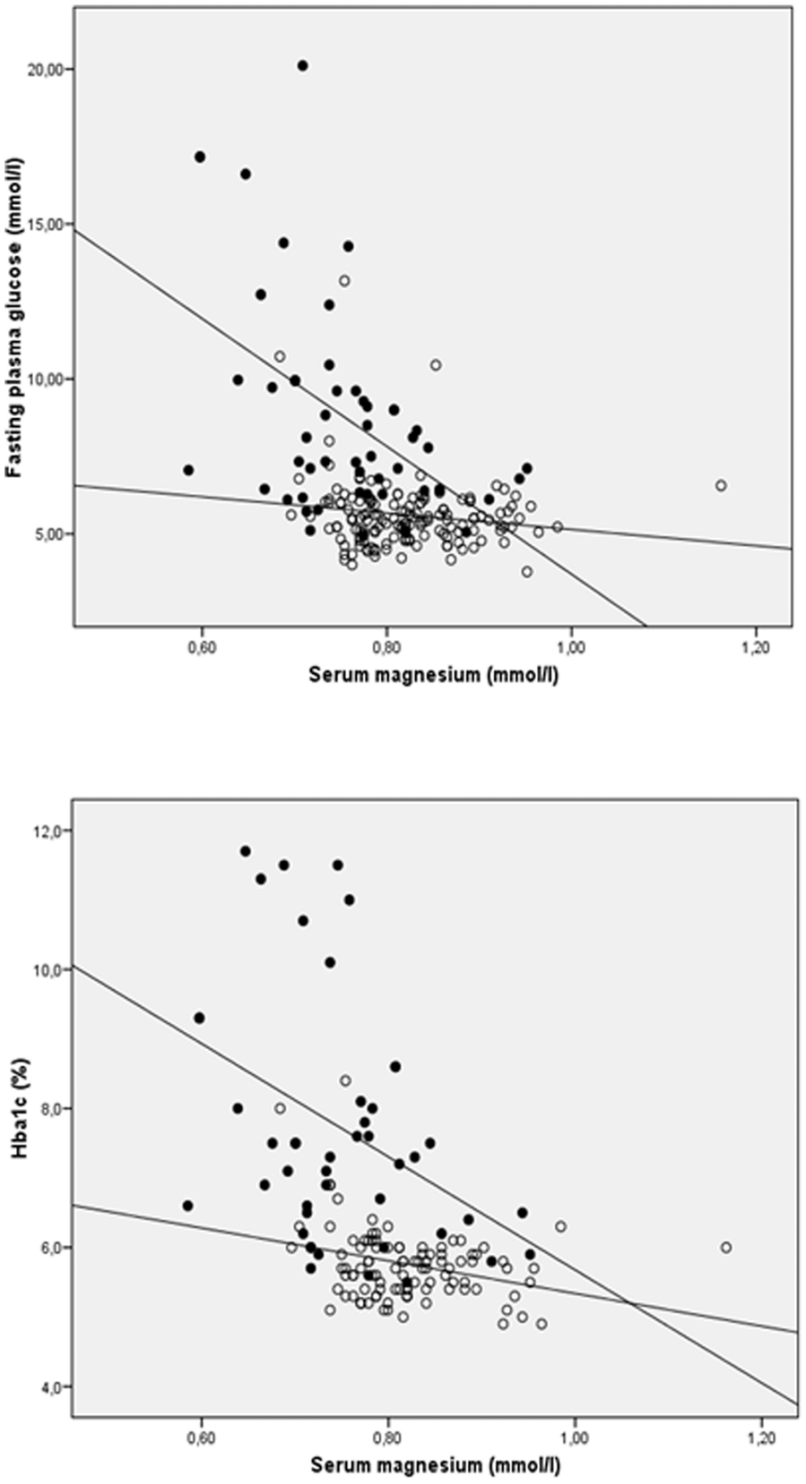
Linear correlations of fasting plasma glucose and HbA1c with serum magnesium in the whole population from the cross sectional case-control study. White circles, non-diabetic patients (fasting plasma glucose: r = −0.155, p = 0.059; HbA1c: r = −0.302, p = 0.002); black circles, type 2 diabetic patients (fasting plasma glucose: r = −0.484, p<0.001; HbA1c: r = −0.389, p = 0.010).

Univariate analysis showed that in the whole population, as well as in diabetic patients, serum magnesium negatively correlated with fasting plasma glucose (r = −0.443, p<0.001 and r = −0.484, p<0.001, respectively) and HbA1c (r = −0.470, p<0.001 and r = −0.389, p = 0.010, respectively) (Figure 1). By contrast, the negative correlation observed between serum magnesium and both HOMA-IR (r = −0.182, p = 0.015) and BMI (r = −0.142, p = 0.046) disappeared when type 2 diabetic subjects were analyzed separately (r = −0.101, p = 0.525 and r = −0.231, p = 0.106, respectively).

Multiple linear regression analyses showed that both HbA1c (in the whole population) and fasting plasma glucose (in type 2 diabetic subjects) were independently related to serum magnesium (Table S1).

### Interventional study: Surgical induced weight loss study

The subset of 120 subjects who underwent RYGBP showed similar baseline characteristics to those observed in the entire population (Table 2). Diabetic patients showed lower serum magnesium concentrations than non-diabetic patients [0.77±0.07 vs. 0.82±0.07 mmol/L; mean difference −0.04 (95% CI −0.08 to −0.02); p = 0.001].

**Table 2:** The main clinical features and laboratory data of the study population underwent RYGBP according to the presence of type 2 diabetes.

	Type 2 Diabetes	Non-diabetic	Mean difference (95%CI)	p
**n**	40	80	-	-
**Women, n (%)**	30 (75.0)	62 (77.5)	-	0.820
**Age (years)**	45.78±8.62	45.18±8.36	0.60 (−2.64 to 3.84)	0.714
**BMI (Kg/m^2^)**	45.75±6.01	46.09±5.18	−0.33 (−2.43 to 1.76)	0.751
**Magnesium (mmol/L)**	0.77±0.07	0.82±0.07	−0.04 (−0.08 to −0.02)	0.001
**Mg≤0.75 mmol/L, n (%)**	18 (45.00)	14 (17.5)	-	0.006
**Fasting plasma glucose (mmol/L)**	8.96±3.61	5.74±1.19	3.21 (2.03 to 4.39)	<0.001
**HbA1c (%)**	7.99±1.82	5.81±0.61	2.17 (1.50 to 2.84)	<0.001
**Fasting insulin (mU/L)**	23.98±14.84	19.09±9.32	4.89 (−1.55 to 11.32)	0.131
**HOMA-IR** *	7.23 (2.24–38.88)	4.22 (1.17–13.85)	-	0.005
**25(OH)D (nmol/L)**	52.35±35.65	52.10±26.90	0.02 (−12.45 to 12.92)	0.971
**iPTH (ng/mL)**	65.44±28.32	70.38±23.03	−4.94 (−16.28 to 6.38)	0.388

Data are mean ± SD, median (range), and percentage. Mg: serum magnesium; BMI: body mass index; HOMA-IR: insulin resistance measured by the homeostasis model assessment; 25(OH)D: 25-hydroxy vitamin D; iPTH: intact parathyroid hormone.

*: Restricted to individuals without current hypoglycaemic medication.

Three months after RYGBP, serum magnesium had significantly increased in those patients in whom diabetes was resolved [0.78±0.07 vs. 0.83±0.05 mmol/L; mean difference 0.05 (95% CI 0.02 to 0.08); p = 0.001] (Table 3). By contrast no significant changes were observed in those patients in whom diabetes was unresolved or in those non-diabetic obese subjects. This effect can not be attributed to weight loss because excess body weight loss (EBWL) was the same in both groups. These findings were maintained in the analyses performed at 6 months of follow-up (Table 3). In addition, no significant differences in baseline serum magnesium concentrations were observed between obese diabetic patients who resolved and not-resolved their diabetes after 6 month of follow up [0.78±0.06 vs. 0.74±0.09 mmol/L; mean difference 0.03 (−0.01 to 0.09); p = 0.152].

**Table 3:** Three and 6-months evolution after RYGBP of serum magnesium concentrations in non-diabetic subjects, and both type 2 diabetic obese patients who resolve and not resolve their diabetes.

	Baseline magnesium(mmol/L)	Follow-up magnesium(mmol/L)	p
**3-months**			
**T2D resolved (n = 21)**	0.78±0.07	0.83±0.05	0.001
**T2D not resolved (n = 19)**	0.76±0.09	0.78±0.09	0.136
**Non diabetic (n = 80)**	0.82±0.07	0.80±0.06	0.456
**6-months**			
**T2D resolved (n = 25)**	0.77±0.06	0.83±0.04	<0.001
**T2D not resolved (n = 15)**	0.77±0.09	0.81±0.07	0.116
**Non diabetic (n = 80)**	0.82±0.07	0.82±0.07	0.931

No differences in EBWL (excess body weight loss) was observed between type 2 diabetic subjects who resolved and not resolved their diabetes at 3-months (38.39±8.10 *vs.* 35.38±14.69%, p = 0.480), nor 6-months (50.87±12.05 *vs.* 48.77±17.27%; p = 0.687) of follow up.

Multiple linear regression analyses showed both in the whole population as in the subgroup of diabetic subjects that absolute changes in HbA1c were independently related to serum magnesium changes at 6 months, and explained 24.7% and 23.2% of the magnesium variation, respectively (Table S2).

No differences in iPTH between patients who resolved and did not resolve their diabetes were observed after 3-months and 6 months of follow-up (80.42±35.56 vs. 80.99±24.05 ng/mL, p = 0.956, and 86.99±44.33 vs. 93.86±27.18 ng/mL, p = 0.604; respectively). In addition, no correlation between serum magnesium and iPTH was observed at 3-months (r = 0.070, p = 0.500) or 6-months (r = −0.034, p = 0.755) after RYGBP. Similarly, no differences in 25(OH)D between patients who resolved or did not resolve their diabetes were observed after 3-months (46.02±25.67 *vs.* 52.42±21.60 nmol/L, p = 0.425) or 6-months (37.77±20.92 *vs.* 33.67±17.3 nmol/L, p = 0.529) of follow-up. Moreover, no correlation between serum magnesium and 25(OH)D was observed at 3-months (r = 0.020, p = 0.831) or 6-months (r = 0.128, p = 0.195) after bariatric surgery.

Two non-diabetic patients required early reoperation for treatment of gastrointestinal leak at the vertical staple-line portion of the gastric pouch. Two more patients (one diabetic and one non-diabetic subject) suffered recurring nausea and vomiting, without evidence of stomal stenosis, which resolved spontaneously after the second month of follow-up. No patient suffered from diarrhoea.

## Discussion

In the present study we provide first evidence that T2DM is the main factor accounting for the low serum magnesium levels found in morbidly obese subjects. In fact, the percentage of subjects with a serum magnesium concentration lower than 0.75 mmol/L was three fold higher in diabetic patients in comparison with non-diabetic subjects. In addition, as previously described [5], we have found a relationship between the degree of blood glucose control (fasting plasma glucose and HbA1c) and magnesium serum concentration, and fasting plasma glucose and HbA1c were independently related to magnesium concentration in multiple linear regression analysis. Furthermore, in the subset of patients who underwent bariatric surgery, serum magnesium increased only in type 2 diabetic subjects in whom diabetes was resolved, but remain unchanged in whom it wasn't as well as in non-diabetic subjects. Finally, absolute changes in HbA1c, but not changes in weight loss, independently predicted changes in serum magnesium after bariatric surgery. Taken together, these findings suggest that the lower levels of magnesium reported in obese subjects are related to the presence of diabetes and glycemic control rather than obesity itself.

The magnesium homeostasis is tightly regulated and depends on the balance between intestinal absorption and renal excretion [16]. In addition, genetic determinants and sex hormones can also modulate serum magnesium levels [17]–[21]. The mechanisms by which T2DM could lead to low serum magnesium levels remain to be fully understood. It has been suggested that it may result from enhanced renal magnesium excretion. Because insulin has been related to magnesium reabsortion at the thick ascending limb of the loop of Henle, insulin deficiency or resistance can promote magnesium wasting at this nephron segment [7], [10]. In addition, hyperglycemia and glycosuria may also interfere with renal magnesium handling, mainly by reducing the tubular reabsorption of the cation. In this regard, McNair *et al* showed that hypermagnesiuria occurred in 55% of insulin-treated diabetic out-patients, and correlated inversely with fasting blood glucose and the urinary glucose excretion rate [22]. Thus, although magnesium excretion was not measured in the present study, an increased urinary magnesium loss could be invoked to explain the low concentration of serum magnesium observed in morbidly obese patients with T2DM. Other potential causes of lower serum magnesium in obese type 2 diabetic patients include reduced intestinal magnesium absorption secondary to higher fat intake and lower fiber intake [23], [24], and diabetic autonomic neuropathy that may reduce oral intake and gastrointestinal absorption [7], [10]. However, a significant role of these mechanisms in accounting for the difference in serum magnesium concentration between diabetic and non-diabetic patients included in the present study is very unlikely. Insulin may induce a shift of magnesium from the plasma to the erythrocytes and smooth muscle cells, both in vivo and in vitro, helping to explain the abnormalities in magnesium circulating levels frequently reported in diabetic patients [25]. However, the lack of difference in serum magnesium levels between type 2 diabetic patients treated with insulin or oral agents suggests that this is not a relevant mechanism, at least in the patients included in the present study.

Low circulating magnesium levels have been related to elevated blood pressure, atherogenic dyslipidemia, impaired clotting, increased inflammatory burden, oxidative stress, carotid wall thickness and coronary heart disease [26], [27]. Therefore, hypomagnesemia could be considered an additional source of cardiovascular risk in T2DM. In addition, hypomagnesemia has been implicated in adversely affecting diabetic complications [28], [29]. Furthermore, it has recently been shown that low serum magnesium concentrations increase the risk of all-cause mortality in T2DM [30]. Obviously, the presence of obesity might increase cardiovascular risk and the mortality rate associated with low magnesium levels.

Apart from diabetic complications and the mortality rate, hypomagnesemia could influence both insulin secretion and insulin action. In this regard it has been suggested that reduced intracellular magnesium concentrations result in an altered cellular glucose transport, a defective tyrosine-kinase activity, post-receptor impairment in insulin action by influencing intracellular signaling and processing, and reduced pancreatic insulin secretion [7], [31]. In addition, chronic magnesium deficiency has also been associated with elevated concentrations of TNF-alpha, and this fact may also contribute to post-receptor insulin resistance [32]. Therefore, T2DM could facilitate low serum magnesium levels and this could in turn worsen glycemic control of diabetes, thus establishing a vicious circle that could lead to a progressive impairment in metabolic control and more risk of diabetic complications. Our results suggest that this vicious circle could be broken by the optimization of metabolic control. In addition, as cereal fiber and magnesium intake have been associated with a reduced risk of T2DM independent of age, sex, and lifestyle risk factors, it also seems prudent to recommend an increased consumption of magnesium-rich foods such as whole grains, beans, nuts and green leafy vegetables in patients with diabetes mellitus who have proven hypomagnesemia [24], [33].

The close relationship between hypomagnesemia and T2DM is reinforced when the evolution of both disorders is evaluated after surgical mediated weight loss. In the present study we provide first evidence that only obese patients who resolved T2DM through the RYGBP achieved a progressive normalization of serum magnesium concentrations. These results can not be attributed to magnesium biological variation and/or analytical imprecision [34]. In addition, we have found that changes in serum magnesium inversely correlated with decreases in both fasting plasma glucose and HbA1c. In addition, absolute changes in HbA1c contributed independently to magnesium changes in multiple linear regression analysis both in the whole population as in diabetic patients, suggesting that metabolic improvement after bariatric surgery is clearly involved in these changes. Limited data about the evolution of serum magnesium after bariatric surgery exist. Although malabsorptive procedures give rise to severe hypomagnesemia, Johansson *et al* have recently communicated an increase in magnesium concentration in non-diabetic morbidly obese patients who underwent RYGBP [14]. This data is in accordance with our results, where serum magnesium concentrations in non-diabetic subjects also increased, but in a smaller magnitude than in diabetic patients. Given that both iPTH and 25(OH)D influence serum magnesium levels, these parameters should be taken into account in the analysis of the results. In this regard serum levels of iPTH and 25(OH)D were closely similar during follow-up in patients in whom diabetes was resolved in comparison with those in whom diabetes was not resolved. In addition, we did not find any correlation between either iPTH or 25(OH)D and serum magnesium levels.

There are some potential limitations that should be taken into account in evaluating the results of our study. First, we did not measure intracellular magnesium content, a more sensitive indicator of magnesium balance [35]. Nevertheless, although approximately only 1% of whole-body magnesium is found extracellularly, serum magnesium exhibits a good correlation with intracellular magnesium measured by nuclear magnetic resonance spectroscopy [36]. Second, we have not specifically evaluated dietary magnesium daily intake. However, given that subjects receiving magnesium supplementation were carefully excluded this possibility is very unlikely. In addition, the same diet was indicated in the subset of patients who underwent bariatric surgery. Finally, only a selected population of morbidly obese subjects was included. Although our results and the possible mechanisms involved in the negative effect of T2DM on magnesium levels might also be transferable to less obese individuals, further studies in these populations are required.

In conclusion, diabetes is the main factor accounting for the low serum magnesium levels found in morbidly obese subjects and it is clearly related to the degree of glycaemic control. Since low serum magnesium levels are an independent cardiovascular risk factor and favour diabetic complications, the normalization of serum magnesium levels is another good reason to recommending the optimization of blood glucose in T2DM.

## Supporting Information

Table S1 Multiple linear regression analysis to explore variables independently related to baseline serum magnesium.
(DOC)

Table S2 Multiple linear regression analysis to explore variables independently related to changes in serum magnesium at 6-months in subjects who underwent RYGBP.
(DOC)
