# Supplementary material for: Diabetes Is the Main Factor Accounting for Hypomagnesemia in Obese Subjects
Source: PLoS One. 2012 Jan 24;7(1):e30599. doi: 10.1371/journal.pone.0030599 (PMC3265490; doi:10.1371/journal.pone.0030599)
Supplement: Table S1 — Multiple linear regression analysis to explore variables independently related to baseline serum magnesium. (DOC) [file pone.0030599.s001.doc]

**Table S1.** Multiple linear regression analysis to explore variables independently related to baseline serum magnesium.

|  | **Baseline serum magnesium** | | | |
| --- | --- | --- | --- | --- |
|  | **All patients** | | **Type 2 diabetes** | |
|  | **beta** | **p** | **beta** | **p** |
| **Fasting glucose** | -0.186 | 0.199 | -0.509 | 0.002 |
| **HbA1c** | -0.435 | <0.001 | -0.040 | -0.851 |
| **BMI** | -0.163 | 0.039 | -0.120 | 0.465 |
| **Age** | 0.120 | 0.132 | 0.127 | 0.407 |
| **HOMA-IR (log)** | 0.076 | 0.422 | 0.255 | 0.185 |
|  | **R^2^=0.227** |  | **R^2^=0.259** |  |
