# Supplementary material for: Diabetes Is the Main Factor Accounting for Hypomagnesemia in Obese Subjects
Source: PLoS One. 2012 Jan 24;7(1):e30599. doi: 10.1371/journal.pone.0030599 (PMC3265490; doi:10.1371/journal.pone.0030599)
Supplement: Table S2 — Multiple linear regression analysis to explore variables independently related to changes in serum magnesium at 6-months in subjects who underwent RYGBP. (DOC) [file pone.0030599.s002.doc]

**Table S2.** Multiple linear regression analysis to explore variables independently related to changes in serum magnesium at 6-months in subjects who underwent RYGBP.

|  | **Δ serum magnesium at 6-month after GBPYR** | | | |
| --- | --- | --- | --- | --- |
|  | **All patients** | | **Type 2 diabetes** | |
|  | **beta** | **p** | **beta** | **p** |
| **Δ Fasting glucose** | 0.047 | 0.762 | 0.075 | 0.692 |
| **Δ HbA1c** | -0.497 | <0.001 | -0.482 | 0.007 |
| **BMI** | 0.027 | 0.835 | 0.031 | 0.855 |
| **Age** | -0.069 | 0.595 | -0.272 | 0.104 |
|  | **R^2^=0.247** |  | **R^2^=0.232** |  |
